# CRISPR/Cas9-mediated editing of barley lipoxygenase genes promotes grain fatty acid accumulation and storability

**DOI:** 10.1080/21645698.2025.2523069

**Published:** 2025-06-26

**Authors:** Zhanghui Zeng, Huiling Wang, Yingjie Luo, Wenjun Chen, Mingrui Xu, Haonan Wei, Zhehao Chen, Taihe Xiang, Lilin Wang, Ning Han, Xiaoping Huang, Hongwu Bian

**Affiliations:** a College of Life and Environmental Sciences, Hangzhou Normal University, Hangzhou, Zhejiang, China; b Key Laboratory for Cell and Gene Engineering of Zhejiang Province, College of Life Sciences, Zhejiang University, Hangzhou, Zhejiang, China

**Keywords:** Artificial aging, barley, CRISPR/Cas9, lipoxygenase (LOX), storability

## Abstract

Plant lipoxygenases (LOXs) catalyze the oxidation of polyunsaturated fatty acids, which can adversely affect grain storability. Although the genetic engineering of *LOXs* holds great potential for improving grain storage quality, this approach remains largely unexplored in barley. In this study, we identified five *LOX* genes in the barley genome: *HvLOXA*, *HvLOXB*, and *HvLOXC1–3*. *HvLOXC1* exhibited the highest expression in early developing grains, roots, and shoots; *HvLOXA* was predominantly expressed in embryos, whereas *HvLOXB* and *HvLOXC3* were weakly expressed across tissues. Transgene-free homozygous barley mutants of *loxB*, *loxC1*, and *loxAloxC1* were generated using CRISPR/Cas9-mediated genome editing. Compared to the wild-type, all mutants displayed normal plant height, tiller number, and grain size, although the *loxC1* and *loxAloxC1* mutants exhibited significantly lower thousand grain weights. Notably, the total LOX activity in mature grains decreased by 36–42% in *loxC1* mutants and by 94% in *loxAloxC1* mutants, with no significant change observed in *loxB* mutants. Additionally, the *loxAloxC1* double mutants had a significantly lower malondialdehyde content and accumulated 10–21% more fatty acids than the wild-type. Artificial aging treatment experiments revealed that *loxAloxC1* mutants had enhanced grain storability, demonstrated by significantly higher germination rates, reduced lipid peroxidation, and improved seedling growth. Our findings highlight that the targeted knockout of *LOX* genes, particularly the double mutation of *HvLOXA* and *HvLOXC1*, represents a promising genetic strategy for improving grain storability and nutritional value in barley.

## Introduction

Lipoxygenase (LOX), widely present in plants, animals, and fungi, is a non-heme iron-containing dioxygenase that catalyzes the addition of oxygen molecules to polyunsaturated fatty acids (PUFAs).^1^ PUFAs, including linoleic acid, linolenic acid, palmitic acid, and arachidonic acid, are oxidized to unsaturated fatty acid hydroperoxides, which subsequently yield various oxylipins and their derivatives, such as jasmonates, green leaf volatiles, death acids, and divinyl ethers.^2,3^ These LOX-derived metabolites play crucial roles in multiple physiological progresses, including plant growth and development, grain germination, signal transduction, stress response, and defense against diseases and insects.^2–6^ However, LOX-catalyzed lipid peroxidation adversely affects grain quality, leading to reduced grain viability and vigor, as well as deterioration of nutritional value.

Higher plants typically contain a complex family of *LOX* genes, many of which exhibit different expression profiles. For example, six *LOXs* have been identified in *Arabidopsis*, among them, *AtLOX2* is highly expressed in leaves, which is upregulated by wound-induced jasmonic acid,^7^ whereas *AtLOX6* displays overall low expression but also contributes to the fast accumulation of jasmonates in wounded leaves and roots.^8,9^ In cotton, 64 putative *LOX* genes were identified, most of which were expressed in vegetative tissues, whereas some genes were highly expressed in specific tissues, such as *GhLOX17* and *GhLOX19* in stigma, *GhLOX14* in fibers, and *GhLOX6* in seeds.^10^ Although *LOX* genes have been systematically identified in various plant species, they are not well understood in barley (*Hordeum vulgare* L.), the fourth most abundant cereal crop in the world. To date, only three single-copy genes–*HvLOXA*, *HvLOXB* and *HvLOXC*–have been identified and described in barley.^11,12^ Transcripts corresponding to these *LOX* genes mainly accumulate in grains and seedlings, whereas no lipoxygenase mRNA has been detected in the aleurone layer of germinating grain in barley.^11^ Despite molecular characterization and expression analyses, the biological functions of these genes in barley have not been well elucidated.

Extensive studies have established a direct correlation between LOX activity and grain viability. Under normal germination conditions, *OsLOX2* overexpression in rice significantly enhances grain germination, whereas RNAi-mediated *OsLOX2* suppression delays this process.^13^ CRISPR/Cas9 knockout of *OsLOX1* or *OsLOX10* reduces the germination rate^14,15^ or has no effect on germination.^16^ However, neither TALEN-induced *OsLOX3* mutagenesis^17^ nor antisense-mediated *OsLOX3* suppression^18^ affects germination efficiency, although certain *OsLOX3*-overexpressing lines exhibit modest but statistically significant reductions in germination capacity relative to wild-type controls.^19^ Both prolonged conventional storage and short-term accelerated aging under high-temperature/humidity conditions generate reactive oxygen species, malondialdehyde (MDA), and 4-hydroxynonenal, causing cellular protein damage and cytotoxic product formation, ultimately compromising grain viability.^1,20–23^ Following artificial aging, the knockout or reduced expression of *OsLOX1-3*, and *OsLOX10* enhances grain germination performance and longevity.^14,16–18^ Conversely, the transgenic overexpression of *OsLOX2* and *OsLOX3* in rice reduces grain vigor.^13,19^ Collectively, these findings indicate functional redundancy among *LOX* family members during grain aging and reveal a complex regulatory network governing *LOX*-mediated grain viability. Reduced or absent *LOX* gene expression can mitigate lipid peroxidation, protect proteins to preserve membrane system integrity, and/or modulate jasmonic acid signaling pathways, thereby enhancing cereal crop tolerance to abiotic stresses.^8,9,14,24^ In addition to affecting grain viability, alterations in LOX activity significantly influence grain nutritional quality. LOX3-null rice variety has less stale flavor during storage than normal LOX3 rice,^1,25,26^ while RNAi-mediated *LOX* suppression in common wheat demonstrates extended grain longevity, improved flour color stability, and elevated fatty acid content following artificial aging treatments.^4,20^ While traditional breeding has successfully developed low-LOX barley varieties with superior brewing characteristics,^27–29^ modern genetic approaches, including gene silencing and editing, now enable more precise grain quality improvements. The CRISPR/Cas9 system has emerged as an ideal precision breeding tool that can generate transgene-free homozygous mutants through genetic segregation.^30,31^ However, because of the well-documented recalcitrance of barley to genetic transformation, the development of genetically edited low-LOX barley lines has not been reported to date.

Recently, global barley production has exceeded 145 million metric tons annually (USDA data; https://www.fas.usda.gov/data/world-agricultural-production). Despite its nutritional value, the storage stability of barley grain remains a critical challenge and requires urgent improvement. In this study, we successfully created transgene-free homozygous *loxB*, *loxC1* and *loxAloxC1* mutants using CRISPR/Cas9-enabled genome editing for genetic and developmental analyses of barley. Plant phenotypic analysis, germination rate statistics, LOX activity, total protein and fat, and MDA content determination, together with fatty acid analysis of grains, showed that the targeted knockout of *HvLOXC1*, especially double mutation in *HvLOXA* and *HvLOXC1* of barley, exhibited better grain storability and higher fatty acid content than the wild-type control, which suggests an effective strategy to improve grain storability and nutritional value of barley grains, as well as for other important agricultural varieties.

## Materials and Methods

### Plant Material and Barley Transformation

Donor plants of the spring barley (*H. vulgare* L. “Golden Promise”) were grown under natural conditions in the experimental fields of Hangzhou Normal University, Zhejiang Province, China. Immature scutella, approximately 1.5 mm in size, were dissected from caryopses that were harvested 15‒20 days after pollination and used as explants for *Agrobacterium-mediated* barley transformation according to a previously detailed protocol.^32^ Transgenic calluses, induced from infected immature scutella on media containing 50 mg L^−1^ hygromycin (Cat.60224ES03, YEASEN, China) for 6‒8 weeks, were then selected for bud and root differentiation and plant regeneration. Regenerated seedlings were grown in a growth chamber with 16 h photoperiod and 23 °C day/20 °C night for at least 50 days and then were transferred to a glasshouse until maturity under natural light.

### Phylogenetic Analysis

The LOX amino acid sequences (Gene ID shown in Table S1) including barley (*H. vulgare*), wheat (*Triticum aestivum*), maize (*Zea mays*), rice (*Oryza sativa*) and Arabidopsis (*Arabidopsis thaliana*) were retrieved from the Ensembl Plants database (https://plants.ensembl.org/index.html.), NCBI (https://www.ncbi.nlm.nih.gov/.) or UniProtKB database (https://www.uniprot.org/help/uniprotkb) and used to construct a phylogenetic tree. Multiple sequence alignments were performed using ClustalW, and subsequent phylogenetic analyses were performed using the maximum likelihood method in MEGA7.

### RNA Extraction and RT-qPCR Analysis

Various tissue samples from barley (*H. vulgare* L. “Golden Promise”) were collected at specific developmental stages, including germinating grains (1 day after germination (DAG)), roots (3/6/8 DAG), shoots (3/6 DAG), leaves (8 DAG), spikes (4 cm length), developing grains (1/3/5/7 days after pollination (DAP)), embryos (6 DAG and 18 DAP), and endosperm (18 DAP). All samples were immediately flash-frozen in liquid nitrogen and ground into a fine powder using a grinding machine (Jingxin, Shanghai, China). Total RNA was extracted using the TRIzol reagent (Invitrogen, Carlsbad, CA, USA). Subsequently, 1 µg of RNA was treated with a gDNA remover kit and reverse transcribed into cDNA using ReverTra Ace qPCR RT Master Mix (Toyobo, Kyoto, Japan) in a 20 µL reaction volume, following the manufacturer’s instructions. The resulting cDNA was diluted 10 times and used for RT-qPCR on a CFX 96 Real-Time System (Bio-Rad, Hercules, CA, USA) using the SYBR PrimeScript RT Reagent Kit (Perfect Real Time, TaKaRa, China). Briefly, 10 µL mixtures for RT-qPCR consisted of 4 µL of cDNA, 5 µL of 2× SYBR Premix Ex Taq II, and 0.5 µL of each forward and reverse primers. The PCR program began with an initial denaturation at 95 °C for 5 min, followed by 40 cycles at 95 °C for 10 s, 60 °C for 15 s, 72 °C for 15 s, and a final melting curve performed under default settings. Three biological replicates were conducted for each sample, and relative transcript levels were calculated using the ^ΔΔ^Ct method, with the *HvACTIN* gene as internal control. The primer sequences for all determined genes are listed in Table S2.

### Guide RNA (gRNA) Design and Vector Construction

Based on the gene sequence of barley *HvLOXA* (HORVU.MOREX.r3.4HG0335790), *HvLOXB* (HORVU.MOREX.r3.4HG0335800) and *HvLOXC1* (HORVU.MOREX.r3.5HG0509200) that downloaded from Ensembl Plants, six single gRNAs were designed and synthesized for targeting six distinct protospacer regions using the web tool CRISPR-P (http://cbi.hzau.edu.cn/crispr/).^33^ gRNA1 and gRNA2 were expected to target the third and fourth exons of *HvLOXA*, respectively; gRNA3 and gRNA4 target the third and fourth exons of *HvLOXB*, respectively; and gRNA5 and gRNA6 target the first two exons of *HvLOXC1*. Polycistronic tRNA-gRNA (PTG) editing constructs containing tRNA-gRNA fragments were generated as described by Xie et al.^30^ Briefly, the tRNA-gRNA fragment was first amplified from the pGTR plasmid and then inserted into a binary vector of pRGEB32, which has an *hpt* selection marker, resulting in three PTG editing constructs: *PTG-HvLOXB*/*Cas9* (gRNA3 + gRNA4), *PTG-HvLOXC1*/*Cas9* (gRNA5 + gRNA6), and *PTG-HvLOXAC1*/*Cas9* (gRNA1 + gRNA2 + gRNA5 + gRNA6). The primers used for the DNA constructs are listed in Table S3, and the detailed sequences of the three PTG constructs are provided in Table S4. These constructs were verified by sequencing, and then electroporated into the *Agrobacterium tumefaciens* strain EHA105 for barley genetic transformation.

### Molecular Characterization of Transgenic Plants

To identify transgenic lines, genomic DNA was extracted from the leaves of primary regenerated plantlets using the CTAB method.^34^ The resultant DNA samples were used for PCR to amplify the gRNA/Cas9 transgene using the primer pair pU3-Forward (5′-TGGGTACGTTGGAAACC ACG-3′) and pUBI10-Reverse (5′-GTTTGTTGGTCGCCGT TAGG-3′), and the targeted protospacer regions of *HvLOXA*, *HvLOXB* and *HvLOXC1* with gene-specific primers shown in Table S5. The PCR products were either examined by 1.5% agarose gel electrophoresis or sequenced directly by the Hangzhou Shangya Biology Company (Hangzhou, China). For heterozygotes of the T_1_ generation, PCR products were purified using a DNA Purification Kit (Sangon Biotech, Shanghai, China) and transferred into a pClone007 simple vector (TsingKe Biotech, Beijing, China) for TA cloning. The resulting products were transformed into the *Escherichia coli* strain DH5α, and 5‒10 single colonies were selected for plasmid isolation and DNA sequencing. Potential off-target sites were predicted by aligning individual gRNA sequences against the genome sequence (version V1) of the “Golden promise” variety on the EnsemblPlants online platform (https://plants.ensembl.org/Hordeum_vulgare_goldenpromise/). For each gRNA, to 2‒3 potential off-target sites with high sequence similarity were selected, followed by PCR primer design and DNA sequencing validation of the PCR products. Detailed information on potential off-target site locations, primer sequences, and PCR products can be found in Excel S1.

### Phenotypic Analysis of Transgenic Plants

Phenotypic analysis was performed on both wild-type barley and its Cas9-free homozygous T3 mutant lines, including *HvloxB7–12*, *HvloxB7–42*, *HvloxC2–37*, *HvloxC10–1*, *HvloxAC11–16* and *HvloxAC11–34*. Plants were grown from November to May in 6-inch pots under natural light in a glasshouse. A minimum of 30, five-month-old plants, were used to analyze the plant height and tiller number. Mature grains were analyzed for grain length and width using ImageJ software, with at least 50 grains analyzed per replicate.

### Artificial Ageing Treatment and Germination Analysis

The dried mature grains of wild-type and *loxB*, *loxC1* and *loxAloxC1* mutant lines were incubated in a controlled environmental incubator set at 40 °C and 85% relative humidity in dark, according to Dong et al.^20^ The grains used for each line were 100 g, divided into four equal parts, and then incubated for 0 (untreated grains were used as controls), 5, 10, or 15 days. After aging, the grains were air-dried and restored to their original weight for subsequent investigation of LOX activity, MDA content, and germination rate. The experiments for the grain germination rate analysis for each line were repeated three times, and each replicate contained at least 50 grains. These grains were sown in the 10 cm square petri dishes, and maintained in the growth chamber under dark condition at 23 °C during the day and 20 °C at night. The germination rate was defined as the percentage of germinated grains on the fourth day. For seedling growth performance analysis, only untreated grains and grains subjected to ten days of artificial aging were used. Root and shoot lengths were measured using rulers on the tenth day after germination in 10 cm square petri dishes.

### Measurement of LOX Activity, MDA Contents, and Total Protein and Fat Content

The total LOX activity levels and MDA content in the mature grains of the wild-type and *LOX* mutant lines were determined using the Plant Lipoxygenase (LOX) Activity Assay Kit (Cat. D799791–0050; Sangon Biotech, Shanghai, China), and MDA Content Assay Kit (Cat. D799761–0050; Sangon Biotech, Shanghai, China), respectively. Total protein and fat contents were measured in mature grains collected 10 days after harvest using MetWare (http://www.metware.cn/). At least three independent measurements were performed for each line using separate grain samples.

### Gas chromatography-Mass Spectrometry (GC-MS) for Fatty Acid Detection

The dried mature grains of wild-type barley and *loxB*, *loxC1*, and *loxAloxC1* mutant lines harvested in May 2024 and stored for two months were used for fatty acid analysis. For each line, 3 g of mature grains were ground into a fine powder in liquid nitrogen twice at 50 hz using a fully automatic sample rapid grinder (Jingxin, Shanghai, China), and five separate samples (50 mg each) were used for subsequent fatty acid extraction and GC-MS analysis by MetWare based on the Agilent 8890-7000E platform.

## Results

### Phylogeny and Expression Analysis of Barley Lipoxygenase Genes

**Figure 1.**
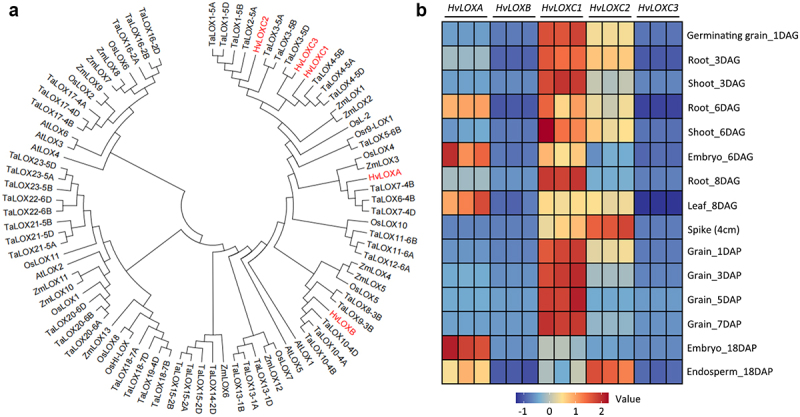
Phylogenetic relationship of LOX amino acid sequences, and transcription patterns of barley *LOX* genes.

Using HvLOXA as the query sequence, we performed genome-wide screening for LOX homologues in barley (*H. vulgare*). Five *LOX* genes were found: *HvLOXA* and *HvLOXB* on chromosome 4, and *HvLOXC1*, *HvLOXC2*, and *HvLOXC3* on chromosome 5 (Table S1). Phylogenetic analysis showed that *HvLOXC1–3* genes were grouped into one cluster, whereas *HvLOXA* was present between *HvLOXB* and the three *HvLOXC* genes (Figure 1a). HvLOXA shares 67.41%, 74.07%, 73.26%, and 68.71% amino acid sequence identity with HvLOXB, HvLOXC1, HvLOXC2, and HvLOXC3, respectively (Figure S1).

(a) LOX proteins of five plant species were used for the construction of the maximum likelihood phylogenetic tree, including barley, wheat, maize, rice and *Arabidopsis*. The protein ID was listed in Table S1. Multiple sequence alignments were performed using the ClustalW program, and subsequent phylogenetic analysis was performed using the maximum likelihood method in the MEGA7 program. The five barley LOXs are highlighted in red. (b) Heat map of RT-qPCR analyses of *HvLOXA*, *HvLOXB*, *HvLOXC1*, *HvLOXC2* and *HvLOXC3* in germination grains (endosperm removed), roots, shoots, leaves, embryos, spike (4 cm spike length), and developing grains. DAG: days after germination, DAP: days after pollination. Three biological replicates were conducted for each sample, and are presented as relative expression levels to the housekeeping gene *HvACTIN*.

RT-qPCR experiments were conducted to investigate gene expression patterns and gain insights into the biological functions of these *LOX* genes. As shown in Figure 1b, *HvLOXA*, *HvLOXC1* and *HvLOXC2* displayed a broader range of expression than *HvLOXB* and *HvLOXC3* in all tested tissues. In germinating grains and seedlings, the expression levels of *HvLOXC1* were generally higher than those of *HvLOXC2*, 3 and 7 times higher in embryos (6 DAG) and roots (8 DAG), respectively. The expression of *HvLOXB* was only one-quarter and six percent of that of *HvLOXA* in roots (8 DAG) and embryos (6 DAG), respectively. After anther pollination, *HvLOXC1* exhibited high expression levels in early grain development (1/3/5/7 DAP) but low expression levels in late grain development (18 DAP) (Figure 1b). The expression levels of *HvLOXC1* were 2, 22, and 7 times higher than those of *HvLOXC2* in grains at 1, 5, and 7 DAP, respectively. Notably, in embryos at 18 DAP, *HvLOXA* exhibited the highest expression level among the five genes, which was 3 folds higher than that of *HvLOXC1*. *HvLOXC3* was weakly expressed in embryos (6 DAG and 18 DAP) and endosperm (18 DAP) and was barely transcribed in other tissues. In summary, these five *LOX* genes displayed clear tissue-specific and developmentally regulated expression patterns during germination and development.

### Design and Delivery of PTG Editing Constructs into Barley Plants

**Figure 2.**
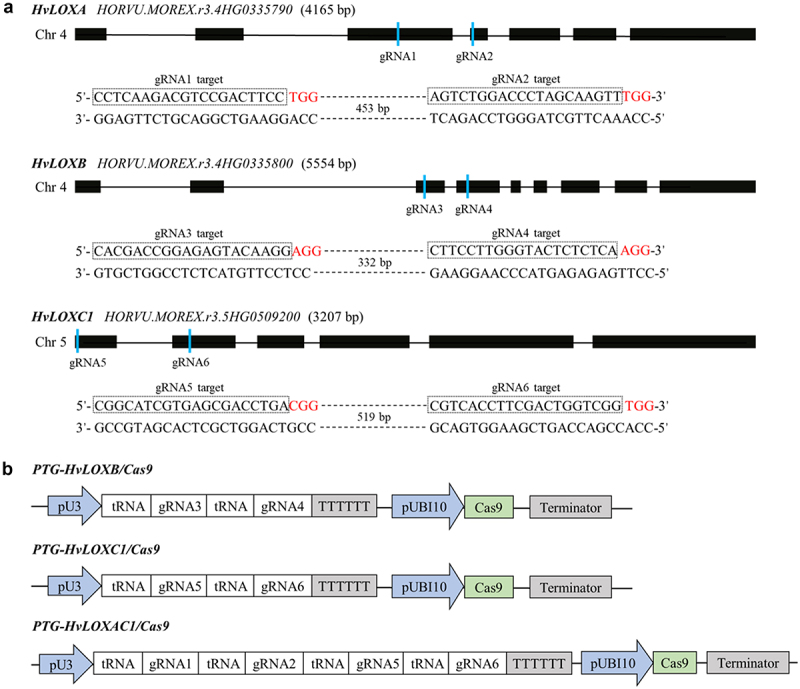
Schematic diagram of *HvLOXA*, *HvLOXB* and *HvLOXC1* genes targeted by six specific gRnas.

Based on their abundant expression in grain tissues (Figure 1), *HvLOXA*, *HvLOXB* and *HvLOXC1* were selected as target genes to create knockout mutants. The genomic sequences of these three genes from the Ensembl Plants database showed that *HvLOXA* (4165 bp) had seven exons and six introns, *HvLOXB* (5554 bp) had nine exons and eight introns, and *HvLOXC1* (3207 bp) had six exons and five introns (Figure 2a). To effectively carry out targeted mutations in these three genes, six single gRNAs were designed to target specific sites in the protein-coding regions with low sequence homology (Figures 2a and S1). As shown in Figure 2b, two or four gRNAs were assembled into a single vector to generate three constructs, *PTG-HvLOXB/Cas9*, *PTG-HvLOXC1/Cas9* and *PTG-HvLOXAC1/Cas9* for *Agrobacterium*-mediated barley transformation. We obtained at least 445, 366, and 655 embryo-derived calli exhibiting hygromycin resistance from the abovementioned constructs. Fifteen primary plantlets of *PTG-HvLOXB/Cas9*, 11 of *PTG-HvLOXC1/Cas9*, and 23 of *PTG-HvLOXAC1/Cas9* were generated from the transformed calli (Table 1).

(a) Gene structure of *HvLOXA* with gRNA1 and gRNA2 targeting sites, *HvLOXB* with gRNA3 and gRNA4 targeting sites, and *HvLOXC1* with gRNA5 and gRNA6 targeting sites. The distance between every two target sites is 453 bp, 332 bp and 519 bp, respectively. Black rectangles indicate protein-coding regions. The PAM, protospacer-adjacent motif, is shown in red. The blue vertical lines indicate the relative location of gRNA targeting sites; the overlined nucleotides indicate the target sequence for each gRNA. (b) Schematic of the tRNA-gRNA fragments inserted into the pRGEB32 vector. TTTTTT: indicate Pol III terminator; pU3, rice U3 snoRNA promoter; pUBI10, rice ubiquitin promoter.

**Table 1.** The mutagenesis frequencies of *HvloxB*, *HvloxC1* and *HvloxAC1* mutants in T_0_ generation.

T-DNA Construct	No. of infected embryos	No. of embryos Anti-*Hpt*	No. of T_0_ plants	No. of mutant T_0_ plants	Mutation rate (%)
*PTG-HvLOXB/Cas9*	800	445	15	8	53.3
*PTG-HvLOXC1/Cas9*	600	366	11	7	63.6
*PTG-HvLOXAC1/Cas9*	1100	655	23	11	47.8

*Anti-Hpt*, hygromycin resistance.

### Characterization and Selection of Cas9-Free Homozygous Barley Mutants

To validate the genetically modified plants, the targeted protospacer regions of *HvLOXA*, *HvLOXB*, and *HvLOXC1* were amplified by PCR for DNA sequencing (5‒10 amplicons per plant; primer sequences are listed in Table S5). The target sequences were aligned against the wild-type DNA sequence, revealing eight mutant lines for *HvLOXB*, seven for *HvLOXC1*, and 11 for *HvLOXA* or *HvLOXC1*. The mutagenesis efficiency among T_0_ regenerated plantlets was 48–64% (Table 1). Since the presence of the Cas9 transgene may induce new mutation types at target sites in the next generation, Cas9-free homozygous mutations for *HvLOXA*, *HvLOXB*, or *HvLOXC1* were screened in T_1_ plants via PCR and DNA sequencing. Among the at least 42 plants in each T_1_ line tested, 0‒20 plants without the Cas9 transgene were obtained from each mutant line (Table 2). There were eight Cas9-free homozygotes for *HvloxB* mutants, including two *loxB-1* plants [1 or 2 bp indel (+T, +A or −TC)], and six *loxB-7* plants [1 bp indel (+A, −A) or 39 bp deletion (−CAAGTTGTCTGACTTCCTTGGGTACTCTCTCAAGGCCAT)]. The number of Cas9-free homozygotes for *loxC11* [3 bp indel (−CCT)], *loxC1–2* [5 bp indel (−GACCT)], and *loxC1–10* [1 bp indel (−C)] lines were 11, 5 and 10, respectively. There were 1‒2 plants of Cas9-free homozygotes for double mutation of *HvLOXA* and *HvLOXC1*, including *loxAC1–6* [21 bp deletion (−AGTTTGGTGACCACACCAGCA) for *HvLOXA*, 3 bp deletion (−GAC) for *HvLOXC1*] and *loxAC1–11* [1 bp indel (−T or −C) for both *HvLOXA* and *HvLOXC1*]. Generally, mutations occurred–3‒4 bp upstream of the protospacer-adjacent motif (PAM) element. Therefore, Cas9-free homozygous single-gene mutants (*HvloxB* and *HvloxC1*) and double-gene mutant (*HvloxAC1*) with different mutation types were obtained from the T_1_ generation.

**Table 2.** Selection and characterization of Cas9-free homozygous barley mutants in T_1_ generation.

T-DNA Construct	T_1_ mutant lines	No. of plants tested	No. of homozygote (Cas9+)	No. of plants without Cas9	No. of heterozygote (Cas9-)	No. of homozygote (Cas9-)	Mutation pattern of homozygote (Cas9-)
*PTG-HvLOXB/Cas9*	*loxB-1*	50		2	0	2	+T/+T; +A/-TC
	*loxB-3*	50	13 (−356bp)	0	0	0	
	*loxB-4*	42	30 (−396bp)	0	0	0	
	*loxB-7*	46		11	4	6	+A/-39bp; +A; -A
*PTG-HvLOXC1/Cas9*	*loxC1–1*	49		20	9	11	−CCT
	*loxC1–2*	50		14	9	5	−GACCT
	*loxC1–10*	50		15	4	10	−C
*PTG-HvLOXAC1/Cas9*	*loxAC1–6*	50		7	6	1	−21bp//-3bp
	*loxAC1–11*	100	4 (-T/+A//-C/+T)	12	8	2	-T//-C

The text before and after the diagonal line indicates the mutation pattern in two gRNA targeting sites; the text before and after the double slash indicates the mutation patterns of *HvLOXA* and *HvLOXC1*, respectively.

### Amino Acid Sequence Analysis of Barley *loxB*, *loxC1* and  Homozygous Mutants

**Figure 3.**
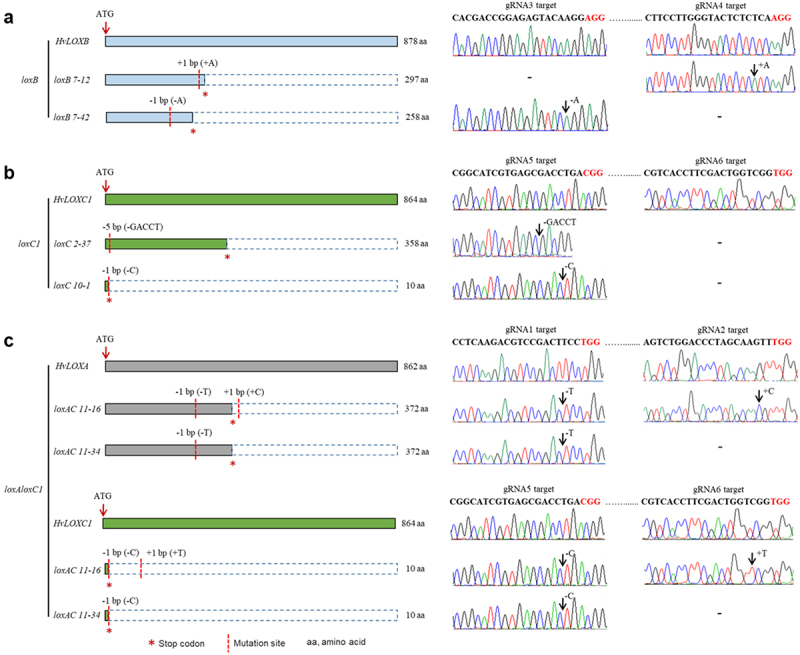
Amino acid sequence analysis and sequencing profiles of gRNA target sites of barley mutants.

DNA sequencing analysis showed that *loxB7–12* and *loxB7–42* carried a single nucleotide insertion or deletion, leading to nonsense mutations at amino acid (aa) 298 (GAC to TGA; Asp to stop) and 259 (CTG to TGA; Leu to stop) of the 878-residue polypeptide (Figure 3a). For *loxC2–37* and *loxC10–1*, nucleotide deletions of GACCT or C resulted in nonsense mutations at amino acids 360 (ACT to TGA, Thr to stop) and 11 (CTG to TGA, Leu to stop) of the 864-residue polypeptide, respectively (Figure 3b). Regarding the targeted protospacer regions of *HvLOXA* and *HvLOXC1* in the double mutants of *loxAC11–16*, a single nucleotide deletion of T or C and an insertion of C or T were found at the gRNA1 and gRNA2 targeting sites, respectively (Figure 3c). In *loxAC11–34* line, a single nucleotide deletion of T or C was found in the gRNA1 targeting sites for both genes. Notably, although the targeted protospacer regions of *HvLOXA* and *HvLOXC1* differed in both double mutants, a single nucleotide deletion resulted in a nonsense mutation at amino acid 373 (ATG to TGA, Met to stop) of the 862-residue polypeptide for *HvLOXA* and 11 (CTG to TGA, Leu to stop) for *HvLOXC1* (Figure 3c). Overall, these deletion and frameshift mutations caused truncations near the N-terminus or led to missense mutations in the respective peptides. In addition, the detection results of potential off-target sites showed that all predicted off-target loci maintained identical sequences to the wild-type, with no detectable mutations (Excel S1), suggesting high specificity of the gene-editing targets. Two Cas9-free homozygous *loxB* lines (*loxB7–12* and *loxB7–42*), two *loxC1* lines (*loxC2–37* and *loxC10–1*), and two *loxAloxC1* mutant lines (*loxAC11–16* and *loxAC11–34*) were used for further studies.

Amino acid sequence analysis and sequencing profiles of gRNA target sites in barley *loxB* (a), *loxC1* (b), and *loxAloxC1* (c) mutants. The red dotted line indicates the mutation site, red asterisk indicates the stop codon, and aa, amino acids.

### Effects of CRISPR/Cas9-Induced Mutations on Plant Phenotype

**Figure 4.**
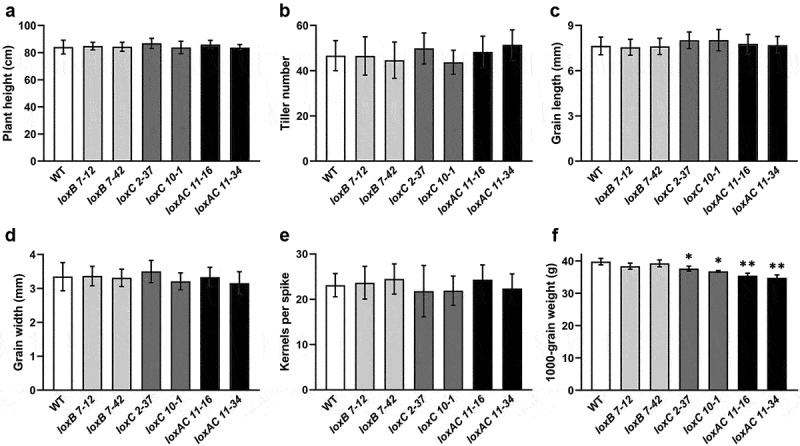
Plant phenotypic observation and grain LOX activity determination of barley *LOX* gene knockout mutants.

To explore the phenotypic impact of *HvLOXA*, *HvLOXB* and *HvLOXC1* knockouts on plant growth, the T_3_ mutants shown in Figure 3 were analyzed. Compared with wild-type plants, no obvious growth differences in plant height, tiller numbers, grain length and width, or kernel number per spike were observed for the *loxB*, *loxC1* and *loxAloxC1* mutants (Figure 4a–e), indicating that the knockout of *HvLOXB*, *HvLOXC1* or both *HvLOXA* and *HvLOXC1* had little effect on plant growth or grain size. Regarding 1000-grain weights, there was no significant difference between the wild-type and *loxB* mutants. However, the 1000-grain weight decreased by 5‒8% in *loxC1* mutants and by 11–13% in *loxAloxC1* mutants compared to that of the wild-type (Figure 4f). These observations indicate that mutations in *HvLOXB* have little effect on plant growth, grain size, or weight, whereas mutations in *HvLOXC1*, especially in combination with *HvLOXA*, significantly decreased grain weight, supporting the significant role of *HvLOXA* and *HvLOXC1* in barley grain development.

(a) Plant height. (b) Tiller number. Five-month-old wild-type (WT) and *LOX* knockout mutant plants were used for the analysis of plant height and tiller number (*n* > 30). (c) Grain length. (d) Grain width. (e) Kernel number per spike (*n* = 30). (f) One thousand-grain weight. Mature grains of Cas9-free, homozygous of T3 mutant lines were used for the analysis of grain length and width (*n* > 50, with ImageJ software), and thousand-grain weight. Asterisks indicate a significant difference between the WT and mutant lines at *p < .05 or **p < .01, as determined by Student’s *t*-test.

### Effects of *LOX* Gene Knockout on LOX Activity, Protein and Fatty Acid Contents in Barley Grains

**Figure 5.**
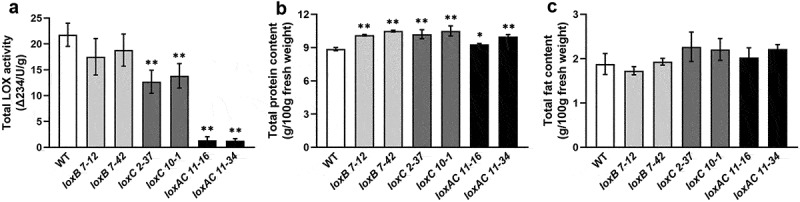
Total LOX activity, protein and fat content in barley grains of *LOX* gene knockout mutants.

To investigate the effects of *HvLOXA*, *HvLOXB* and *HvLOXC1* mutations on LOX activity, the total LOX activity was determined for the flour samples of mature barley grains. The results showed that, compared with the wild-type, the total LOX activity decreased significantly by 36‒42% in *loxC1* mutants and by 94% in *loxAloxC1* mutants, but did not change significantly in *loxB* mutants (Figure 5a). These results are consistent with the 1000-grain weight trait and show that mutations in *HvLOXC1*, particularly the double mutations of *HvLOXA* and *HvLOXC1*, significantly decreased grain LOX activity. We also measured the total protein and fat contents of the mature grains. The results showed that the total protein content was significantly upregulated in all *lox* mutants compared to the wild-type, whereas the total fat content remained unchanged (Figure 5b,c).

Mature grains of Cas9-free homozygous T3 mutant lines were used to analyze total LOX activity (a), protein content (b), and fat content (c). Means and standard deviations of at least three replicates are presented. Asterisks indicate a significant difference between the WT and mutant lines at *p < .05, or **p < .01, as determined by the Student’s *t*-test.

To investigate whether the accumulation of fatty acids is altered in LOX knockout mutants, mature grain samples were analyzed using GC-MS. The palmitic acid (C16:0), stearic acid (18:0), oleic acid (18:1), linoleic acid (18:2), and linolenic acid (18:3) contents were measured (Table 3). Compared to wild-type, no significant difference was observed for the contents of these five fatty acids and their combined amounts in *loxB* mutants. However, the contents of these five fatty acids were generally higher in the grain samples of *loxAloxC1* and *loxC1* mutants than in those of wild-type. The contents of linoleic acid, linolenic acid, palmitic acid, stearic acid and oleic acid in the *loxC1* mutants were slightly increased by up to 8.47%, 9.42%, 11.85%, 7.25% and 3.96%, respectively; the combined amounts of the five fatty acids were 5.60% (*loxC10–1*) and 7.81% (*loxAC2–37*) higher than that of wild-type. The five fatty acids in *loxAloxC1* mutants increased by 7.78‒15.87%, 2.51‒20.67%, 20.74‒41.16%, 11.54‒20.72%, and 5.23‒14.32%, respectively. The combined amounts of these five fatty acids in the two *loxAloxC1* mutants were 10.40% (*loxAC11–16*) and 20.73% (*loxAC11–34*) higher than in wild-type (Table 3). These results indicate that mutations in both *HvLOXA* and *HvLOXC1* have a greater impact on fatty acid content than a single gene mutation in *HvLOXC1*, suggesting that *HvLOXA* might be a more crucial gene regulating fatty acid biosynthesis in barley than *HvLOXC1* alone.

**Table 3.** Evaluation of grain fatty acid contents of wild-type and barley *LOX* gene knockout mutants.

Fatty acid composition (µg/mg)	WT	*loxB7–12*	*loxB7–42*	*loxC2–37*	*loxC10–1*	*loxAC11–16*	*loxAC11–34*
Palmitic acid (C16:0)	1.50 ± 0.05	1.52 ± 0.06 [1.22%]	1.56 ± 0.10 [4.17%]	1.63 ± 0.05 [8.47%]**	1.55 ± 0.07 [3.63%]	1.62 ± 0.05 [7.78%]**	1.74 ± 0.08 [15.87%]***
Stearic acid (18:0)	0.35 ± 0.02	0.36 ± 0.03 [3.90%]	0.36 ± 0.03 [3.98%]	0.38 ± 0.02 [9.42%]*	0.35 ± 0.03 [1.95%]	0.36 ± 0.01 [2.51%]	0.42 ± 0.03 [20.67%]**
Oleic acid (C18:1)	0.50 ± 0.03	0.51 ± 0.01 [2.76%]	0.54 ± 0.05 [7.23%]	0.56 ± 0.01 [11.85%]**	0.57 ± 0.02 [13.37%]**	0.60 ± 0.03 [20.74%]***	0.71 ± 0.03 [41.16%]***
Linoleic acid (C18:2)	3.30 ± 0.11	3.30 ± 0.10 [0.05%]	3.40 ± 0.25 [3.18%]	3.54 ± 0.11 [7.25%]*	3.53 ± 0.15 [6.92%]*	3.68 ± 0.14 [11.54%]**	3.98 ± 0.15 [20.72%]***
Linolenic acid (C18:3)	0.44 ± 0.02	0.46 ± 0.02 [4.61%]	0.48 ± 0.04 [8.88%]	0.46 ± 0.01 [3.96%]	0.43 ± 0.02 [−3.46%]	0.47 ± 0.02 [5.23%]*	0.51 ± 0.02 [14.32%]**
Total fatty acids	6.09 ± 0.22	6.11 ± 0.19 [1.11%]	6.35 ± 0.46 [4.22%]	6.57 ± 0.20 [7.81%]**	6.43 ± 0.25 [5.60%]*	6.72 ± 0.24 [10.40%]**	7.35 ± 0.30 [20.73%]***

Each value (µg/mg grain) was the mean ± standard deviation of five independent grain samples. *, ** and *** indicate statistical difference from wild-type (WT) at *P* < 0.05, 0.01 and 0.001, respectively. Values in square brackets indicate the percentage increase over WT.

### Effects of Artificial Aging on Grain Germination and Seedling Growth of *LOX* Gene Knockout Mutants

**Figure 6.**
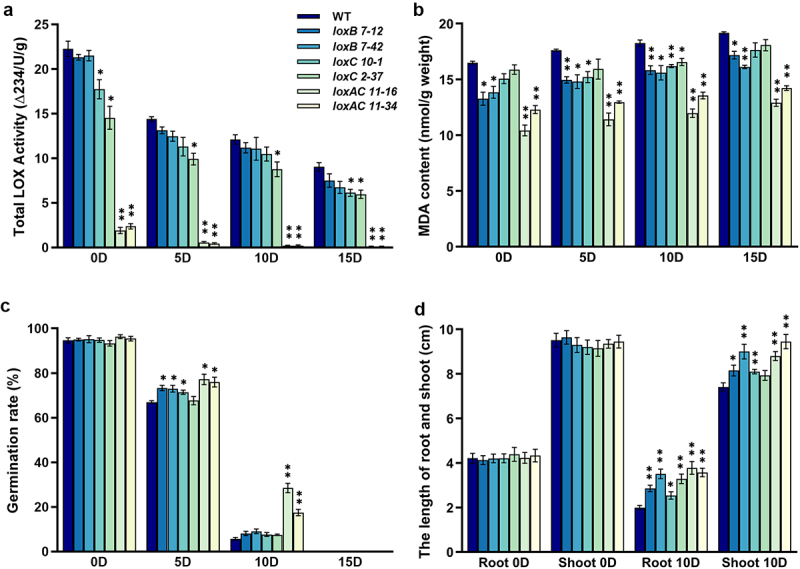
Effects of artificial aging on the grains of barley *LOX* gene knockout mutants.

To investigate whether CRISPR/Cas9-mediated targeted mutagenesis of *HvLOXA*, *HvLOXB* and *HvLOXC1* improved grain storability, artificial aging treatment was conducted. Overall, artificial aging substantially decreased LOX activities in both wild-type and six mutant lines (Figure 6a). During a 15-day artificial aging period, the levels of LOX activities in *loxC1* and *loxAloxC1* mutants were generally much lower than that of wild-type, particularly in *loxAloxC1* mutants whose LOX activity levels dropped almost to zero (Figure 6a). MDA increased gradually during artificial aging in both wild-type and six mutant lines. Again, the MDA content in all *lox* mutants was consistently lower than that in the wild-type, with the percentage reduction ranging from 13‒20% in *loxB* mutants, 4‒14% in *loxC1* mutants, and 25‒37% in *loxAloxC1* mutants (Figure 6b). Before artificial aging, no significant difference was observed in grain germination rate between wild-type and six mutant lines. However, after 5 or 10 days of artificial aging, the germination rates of the two *loxAloxC1* mutant lines were significantly higher than that of wild-type, with an increase by 9–10% and 12–23% in 5- and 10-day, respectively (Figure 6c). After 5-day artificial aging, germination rates of the two *loxB* mutants were increased by 6%, while slightly increased by 4% in *loxC1* mutant (*loxC10–1*), compared to that of the wild-type. After 15-day artificial aging, neither wild-type nor mutant grains germinated (Figure 6c). Moreover, the growth performance of 10-day artificial aging treated grains was investigated. The root and shoot lengths of all mutant lines were much higher than those of the wild-type, indicating that *LOX* gene knockout exhibited much better growth performance than the wild-type (Figure 6d). Taken together, these results suggest that knockout of *LOX* genes, particularly double mutations in *HvLOXA* and *HvLOXC1*, could effectively improve seedling growth after artificial aging.

The mature grains of the wild-type (WT) and six *LOX* gene knockout mutants were subjected to various assays at 0 (untreated control), 5, 10, or 15 days post artificial aging treatment. (a) Total LOX activity (b) MDA content. (c) Germination rate. (d) The length of roots and shoots of the seedling that were germinated from the grains of WT and six *LOX* gene knockout mutants at 0 and 10 days post artificial aging treatment. Data (means ± standard deviation) were derived from at least three independent samples. Asterisks (* or **) indicate a significant difference between the WT and mutants, at *p* < .05 or *p* < .01, respectively, as determined by Student’s *t*-test.

## Discussion

Grain storability is an important agronomic and physiological trait in crop production. To date, little is known about the effects of barley LOXs on grain storability and transgenic plants with reduced LOX activity have not been developed. In the present study, we performed gene expression analyses of five barley *LOX* genes, created *loxB*, *loxC1* and *loxAloxC1* mutants, for further agronomic and physiological analyses before and after artificial aging treatment, and characterized the effects of *LOX* gene mutations in barley.

### Transcript Levels of Five *LOX* Genes Varied in Different Tissues of Barley

Previous studies identified three *LOX* genes in barley: *HvLOXA*, *HvLOXB* and *HvLOXC*. The former two genes are located on chromosome 4, whereas the latter is located on chromosome 7.^11,12^ With the release of barley Pan-genome database,^35^ a total of five *LOX* genes (*HvLOXA*, *HvLOXB*, *HvLOXC* (renamed as *HvLOXC1*), *HvLOXC2*, *HvLOXC3*) were found in this study (Figure 1). We found that *HvLOXC1* is located on chromosome 5 rather than on chromosome 7. *HvLOXC2* and *HvLOXC3* are also located on chromosome 5 (Table S1). These results highlight the importance of barley genomic data for identifying hidden information in DNA sequences. In addition, the expression levels of the five barley *LOX* genes varied among the different barley tissues. *HvLOXC1* was highly expressed in various tissues in both seedlings and early developing grains, while *HvLOXA* exhibited predominate expression in embryos, and *HvLOXB* was weakly expressed in roots and embryos (Figure 1). These results are consistent with previous findings that *LOX* gene transcripts mainly accumulate in grains and seedlings.^11^ *HvLOXB* has a minimal impact on beer quality because of its low expression in barley kernels and ambiguous product specificity.^11^ Although transcriptional activation of *HvLOXB* occurs in barley leaf tissue in response to osmotic stress (sorbitol) and jasmonate signaling,^36^ its biological roles require further clarification. Additionally, *HvLOXC1* showed generally higher expression than that of *HvLOXC2*, whereas *HvLOXC3* exhibited extremely low expression, suggesting that *HvLOXC1* plays a primary role in LOX biosynthesis. Therefore, based on *LOX* gene expression data, *HvLOXA*, *HvLOXB* and *HvLOXC1* were functionally analyzed in barley using CRISPR/Cas9 gene editing.

### Diverse Function of Barley *LOX* Genes in Regulating Grain Development and Storability

Transgenic plants with active gRNA/Cas9 transgenes may generate more mutation types than transgene-free plants in subsequent generations, thereby affecting the genetic phenotype analysis.^31^ Therefore, it is necessary to generate transgene-free plants with fixed targeted mutations. To analyze the differences in gene function among barley *LOX* family members, transgene-free homozygous mutants with varying degrees of LOX activity reduction were obtained using CRISPR/Cas9-mediated technology (Table 2 and Figure 5). All the mutants displayed normal phenotypes similar to the wild-type plants except for the thousand-grain weight, which significantly decreased in *loxC1* and *loxAloxC1* mutants, but not in *loxB* mutants (Figure 4), indicating diverse regulatory roles of *LOX* genes in barley grain formation. Total LOX enzyme activity was substantially reduced by 94% in mature grains of *loxAloxC1* double mutants, and was reduced by approximately 40% in *loxC1* mutants (Figure 5). This suggests that *HvLOXA* may play a more important role than *HvLOXC1* in determining LOX activity in barley grains. Consistent with previous studies, LOX-1 (HvLOXA) contributed almost exclusively to the total LOX activity in quiescent barley grains.^37^ Meanwhile, *HvLOXB*, *HvLOXC2* and *HvLOXC3*, may display a weak contribution to the total LOX activity. In fact, mutation in *HvLOXB* did not result in obvious changes in LOX activity or in plant height and grain size, compared to the wild-type (Figure 4). Overall, LOX activity in various *lox* mutants was consistent with the expression levels of *LOX* homologue genes in barley grains.

Grain storability is a complex process regulated by both genetic and environmental factors such as high humidity, high temperature, and oxidation, as well as by multiple genes associated with grain storability.^22,23,38^ Lipid peroxidation critically impacts grain storage, as MDA, its most mutagenic product, directly reflects cell membrane damage severity.^39^ Both before and after artificial aging treatment with a short period of high temperature and humidity conditions, *loxAloxC1* double mutants presented the largest decline in MDA content and the lowest decrease in germination rate compared to the wild-type (Figure 6). A substantial decrease in LOX activity delays lipid peroxidation and reduces the formation of cytotoxic substances, thus protecting cell structures.^1^ Therefore, mutations in both *HvLOXA* and *HvLOXC1* significantly decreased LOX activity and significantly improved grain longevity and viability after artificial aging. This result is consistent with transgenic studies in other crop species, such as rice, wheat, maize, sorghum, etc.^4,6,13,14,18,20,24,40,41^ By silencing the *LOX* gene in rice and wheat using RNA interference technology, lipid peroxidation can be significantly reduced, thereby reducing the deterioration of grain quality and the decline in nutritional value during storage.^4,20,42^ Notably, *loxAloxC1* double mutant showed significantly improved fatty acid content compared to the wild-type and single gene mutation of *HvLOXC1* (Table 3). Consistent with the weak expression of *HvLOXB* in developing grains (Figure 1), no significant changes in fatty acid content were observed in *loxB* mutants. Previous studies have reported that fatty acids and oxidized triacylglycerols are generally increased in long-term-aged seeds of *Arabidopsis*, leading to decreased germinability.^22^ A similar negatively correlation between germination frequencies and the concentrations of lipid hydrolysis products and oxidized lipids, such as fatty acids, lysophospholipids, and oxidized diacylglycerols, was found by Wiebach et al.^23^ Decreased LOX activity may reduce lipid oxidation and delay the hydrolysis of structural and storage lipids, thereby improving grain viability.

### Application of *loxAloxc1* Double Mutant in Economical Barley Varieties

Barley is a transformation-recalcitrant species and genome editing is often inefficient, labourious, and time-consuming. In this study, only 8–11 regenerated plantlets were obtained from 366–655 embryo-derived calluses with hygromycin resistance, and the mutagenesis efficiency among the T_0_ regenerated plantlets was approximately 50% (Table 1). The entire transgenic process lasts approximately five months. Moreover, the transformation efficiency of barley is greatly influenced by the variety, which is especially difficult for economically important barley cultivars such as Harrington, Hector, and Morex.^43^ In most transformation protocols, the donor plants of the spring barley “Golden Promise” are usually used as model cultivars, but are not considered agriculturally important. Based on this cultivar, we successfully performed targeted mutagenesis of the *LOX* genes in barley via CRISPR/Cas9-mediated genome editing and obtained T1 generation transgene-free homozygous mutants, including single or double gene mutants. After artificial aging, transgene-free homozygous grains of *loxC1* and *loxAloxC1* double mutants exhibited significantly lower LOX activity and MDA content, but a higher grain germination rate and better seedling growth performance (Figures 5,6), indicating the potential application of this approach in improving grain storability in cereal crops. Moreover, *loxAloxC1* double mutant exhibited a more obvious difference from the wild-type than the single gene knockout mutant*s loxC1* or *loxB* in terms of grain LOX activity, lipid peroxidation, and germination rate. Our results suggest that *HvLOXA* is a key gene regulating LOX enzyme activity and grain storability. To obtain a single-gene knockout mutant of *loxA*, a transgene-free *loxAloxC1* double mutant could be used for crossbreeding with superior local economically important varieties or imported germplasm to develop better germplasm resources. *HvLOXA* and *HvLOXC1* are located on different chromosomes and *HvloxA* can be selected via homologous chromosome separation and non-homologous chromosome combinations in subsequent generations. In addition, knockout of *LOX* genes, especially in *loxAloxC1* double mutants, increased the total fatty acid content (Table 3). Fatty acids are a crucial component of total lipids in cereal grains and are closely linked to grain quality and nutritional value.^44,45^ Fatty acids influence various aspects of grain quality such as flavor, aroma, and processing properties. Future studies are needed to investigate the effects of altered lipid accumulation on grain processing quality in these *lox* mutants. Overall, *loxAloxC1* double mutants may be further developed into a potential commercial line with enhanced storability and increased grain fatty acid content.

## Conclusion

In conclusion, we identified five homologous *LOX* genes in the barley genome. *HvLOXA*, *HvLOXC1* and *HvLOXC2* showed distinct expression patterns in different tissues, whereas *HvLOXB* and *HvLOXC3* exhibited very low expression levels. Transgene-free homozygous barley mutants of *loxB*, *loxC1*, and *loxAloxC1* were successfully generated using the CRISPR/Cas9 system. The *loxAloxC1* double mutants reduced most LOX activity and accumulated higher fatty acid content than the wild-type. Artificial aging experiments showed that *loxAloxC1* double mutants exhibited better grain storability, including higher germination rates, lower lipid peroxidation levels, and better seedling growth. This study provides an effective strategy for using *LOX* gene-editing technology to improve the nutritional value and storability of barley grains for future cereal breeding and processing.

## Supplementary Material

Supplemental data_Fig and Tables_20250614.docx

Supplemental data_Excel S1_20250614.xlsx
