# Supplementary material for: CRISPR/Cas9-mediated editing of barley lipoxygenase genes promotes grain fatty acid accumulation and storability
Source: GM Crops Food. 2025 Jun 26;16(1):482–97. doi: 10.1080/21645698.2025.2523069 (PMC12203851; doi:10.1080/21645698.2025.2523069)
Supplement: Supplemental data_Fig and Tables_20250614.docx [file KGMC_A_2523069_SM2618.docx]

**Supplementary data**
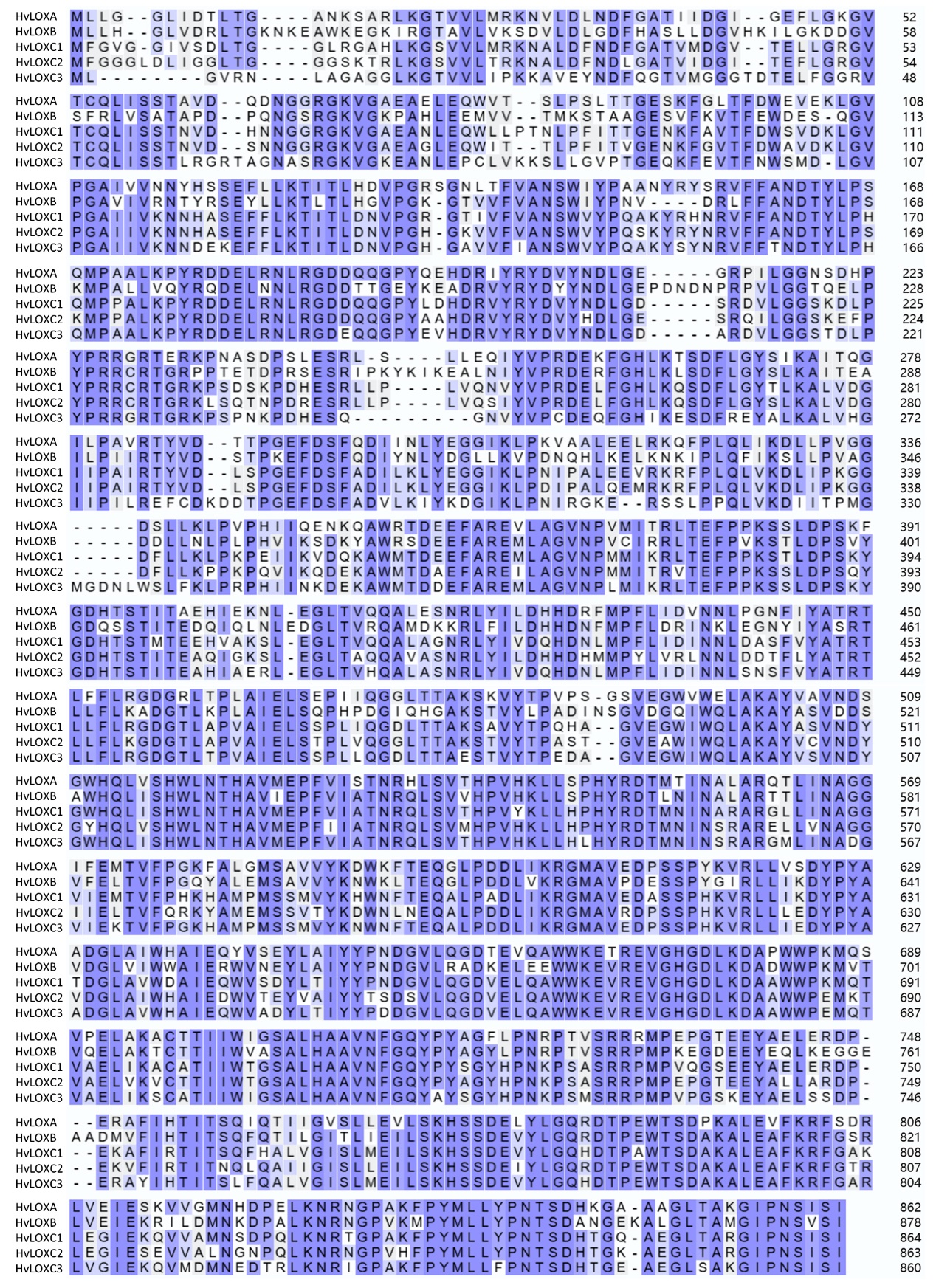


**Fig. S1** Comparison of amino acid sequences for five barley *LOX* genes.

Similarity comparison of amino acid sequences among five barley *LOX* genes. The amino acid sequences were aligned using the Clustal Omega program. The amino acid sequence of HvLOXA shares 67.41%, 74.07%, 73.26%, and 68.71% identity with HvLOXB, HvLOXC1, HvLOXC2, and HvLOXC3, respectively.

**Table S1** Gene ID of the LOX amino acid sequences for barley and other four species.

| **Species name** | **Gene name** | **Gene ID** |
| --- | --- | --- |
| *Hordeum vulgare* | *HvLOXA* | HORVU.MOREX.r3.4HG0335790 |
|  | *HvLOXB* | HORVU.MOREX.r3.4HG0335800 |
|  | *HvLOXC1* | HORVU.MOREX.r3.5HG0509200 |
|  | *HvLOXC2* | HORVU.MOREX.r3.5HG0509210 |
|  | *HvLOXC3* | HORVU.MOREX.r3.5HG0529630 |
| *Zea mays* | *ZmLOX1* | Zm00001d042541 |
|  | *ZmLOX2* | Zm00001d042540 |
|  | *ZmLOX3* | Zm00001d033623 |
|  | *ZmLOX4* | Zm00001d033624 |
|  | *ZmLOX5* | Zm00001d013493 |
|  | *ZmLOX6* | Zm00001d002000 |
|  | *ZmLOX7* | Zm00001d025524 |
|  | *ZmLOX8* | Zm00001d003533 |
|  | *ZmLOX9* | Zm00001d027893 |
|  | *ZmLOX10* | Zm00001d053675 |
|  | *ZmLOX11* | Zm00001d015852 |
|  | *ZmLOX12* | Zm00001d041204 |
|  | *ZmLOX13* | Zm00001d031449 |
| *Oryza sativa* | *OsLOX1* | LOC_Os02g10120 |
|  | *OsLOX4* | LOC_Os03g49350 |
|  | *OsLOX2* | LOC_Os03g08220 |
|  | *Osr9-LOX1* | LOC_Os03g49260 |
|  | *OsLOX5* | LOC_Os03g49380 |
|  | *OsL-2* | LOC_Os03g52860 |
|  | *OsLOX6* | LOC_Os04g37430 |
|  | *OsLOX7* | LOC_Os05g23880 |
|  | *OsHI-LOX* | LOC_Os08g39840 |
|  | *OsLOX8* | LOC_Os08g39850 |
|  | *OsLOX10* | LOC_Os11g36719 |
|  | *OsLOX11* | LOC_Os12g37260 |
| *Arabidopsis thaliana* | *AtLOX1* | AT1G55020 |
|  | *AtLOX3* | AT1G17420 |
|  | *AtLOX6* | AT1G67560 |
|  | *AtLOX4* | AT1G72520 |
|  | *AtLOX5* | AT3G22400 |
|  | *AtLOX2* | AT3G45140 |
| *Triticum aestivum* | *TaLOX1-5A* | TraesCS5A02G378900 |
|  | *TaLOX1-5B* | TraesCS5D02G388800 |
|  | *TaLOX1-5D* | TraesCS5A02G379000 |
|  | *TaLOX2-5A* | TraesCS5B02G382600 |
|  | *TaLOX3-5A* | TraesCS5A02G378800 |
|  | *TaLOX3-5B* | TraesCS5B02G382400 |
|  | *TaLOX3-5D* | TraesCS5D02G513100 |
|  | *TaLOX4-5A* | TraesCS5A02G378700 |
|  | *TaLOX4-5B* | TraesCS5B02G382300 |
|  | *TaLOX4-5D* | TraesCS5D02G388700 |
|  | *TaLOX5-6B* | TraesCS6B02G395700 |
|  | *TaLOX6-4B* | TraesCS4B02G037700 |
|  | *TaLOX7-4B* | TraesCS4B02G037900 |
|  | *TaLOX7-4D* | TraesCS4D02G035200 |
|  | *TaLOX8-3B* | TraesCS3B02G602500 |
|  | *TaLOX9-3B* | TraesCS3B02G605700 |
|  | *TaLOX10-4A* | TraesCS4A02G275900 |
|  | *TaLOX10-4B* | TraesCS4B02G037800 |
|  | *TaLOX10-4D* | TraesCS4D02G035100 |
|  | *TaLOX11-6A* | TraesCS6A02G132200 |
|  | *TaLOX11-6B* | TraesCS6B02G160400 |
|  | *TaLOX12-6A* | TraesCS6A02G132500 |
|  | *TaLOX13-1A* | TraesCS1A02G212900 |
|  | *TaLOX13-1B* | TraesCS1B02G226400 |
|  | *TaLOX13-1D* | TraesCS1D02G215800 |
|  | *TaLOX14-2D* | TraesCS2D02G528400 |
|  | *TaLOX15-2A* | TraesCS2A02G525500 |
|  | *TaLOX15-2B* | TraesCS2B02G555400 |
|  | *TaLOX15-2D* | TraesCS2D02G528500 |
|  | *TaLOX16-2A* | TraesCS2A02G315100 |
|  | *TaLOX16-2B* | TraesCS2B02G333600 |
|  | *TaLOX16-2D* | TraesCS2D02G313400 |
|  | *TaLOX17-4A* | TraesCS4A02G009400 |
|  | *TaLOX17-4B* | TraesCS4B02G295200 |
|  | *TaLOX17-4D* | TraesCS4D02G294100 |
|  | *TaLOX18-7A* | TraesCS7A02G246200 |
|  | *TaLOX18-7B* | TraesCS7B02G145200 |
|  | *TaLOX18-7D* | TraesCS7D02G244800 |
|  | *TaLOX19-4D* | TraesCS4D02G347300 |
|  | *TaLOX20-6A* | TraesCS6A02G166000 |
|  | *TaLOX20-6B* | TraesCS6B02G193400 |
|  | *TaLOX20-6D* | TraesCS6D02G154600 |
|  | *TaLOX21-5A* | TraesCS5A02G032800 |
|  | *TaLOX21-5B* | TraesCS5B02G033000 |
|  | *TaLOX21-5D* | TraesCS5D02G041100 |
|  | *TaLOX22-6B* | TraesCS6B02G014400 |
|  | *TaLOX22-6D* | TraesCS6D02G010100 |
|  | *TaLOX23-5A* | TraesCS5A02G007900 |
|  | *TaLOX23-5B* | TraesCS5B02G006500 |
|  | *TaLOX23-5D* | TraesCS5D02G013400 |

**Table S2** Primers for RT-qPCR.

| **Gene** | **Forward primer (5′-3′)** | **Reverse primer (5′-3′)** | **Product size (bp)** |
| --- | --- | --- | --- |
| *HvLOXA* | CGCCACGTACGAAACTTGTC | CGCTTAAAAACGGATGGGGC | 180 |
| *HvLOXB* | TTCTGGGTGGTTGCGGTAAT | TCTCTTGCACTTGTACCGAACT | 226 |
| *HvLOXC1* | AAGAACGCGCTCGACTTCAA | GTTGTTGTGGTCGACGTTGG | 117 |
| *HvLOXC2* | GCGTGAACGACTACGGCTAT | TGCTCGTTGAGGTTCCAGTC | 277 |
| *HvLOXC3* | GTACAACGACTTCCAGGGCA | CTTCTTCACGAGACACGGCT | 172 |
| *HvACTIN* | GCTGAGCGGGAAATTGTAAG | GATCATGGATGGCTGGAAGA | 192 |

**Table S3** Primers used for making PTG editing constructs.

| **Primer ID** | **Sequence (5′-3′)** |
| --- | --- |
| L5AD5-F | CGGGTCTCAGGCAGGATGGGCAGTCTGGGCAACAAAGCACCAGTGG |
| L3AD5-R | TAGGTCTCCAAACGGATGAGCGACAGCAAACAAAAAAAAAAGCACCGACTCG |
| S5AD5-F | CGGGTCTCAGGCAGGATGGGCAGTCTGGGCA |
| S3AD5-R | TAGGTCTCCAAACGGATGAGCGACAGCAAAC |
| *HvLOXA*-gRNA1-F | TAGGTCTCCCGTCCGACTTCCGTTTTAGAGCTAGAA |
| *HvLOXA*-gRNA1-R | CGGGTCTCAGACGTCTTGAGGTGCACCAGCCGGG |
| *HvLOXA*-gRNA2-F | TAGGTCTCCCCCTAGCAAGTTGTTTTAGAGCTAGAA |
| *HvLOXA*-gRNA2-R | CGGGTCTCAAGGGTCCAGACTTGCACCAGCCGGG |
| *HvLOXB*-gRNA3-F | TAGGTCTCCGAGAGTACAAGGGTTTTAGAGCTAGAA |
| *HvLOXB*-gRNA3-R | CGGGTCTCATCTCCGGTCGTGTGCACCAGCCGGG |
| *HvLOXB*-gRNA4-F | TAGGTCTCCGGTACTCTCTCAGTTTTAGAGCTAGAA |
| *HvLOXB*-gRNA4-R | CGGGTCTCATACCCAAGGAAGTGCACCAGCCGGG |
| *HvLOXC1*-gRNA5-F | TAGGTCTCCTGAGCGACCTGAGTTTTAGAGCTAGAA |
| *HvLOXC1*-gRNA5-R | CGGGTCTCACTCACGATGCCGTGCACCAGCCGGG |
| *HvLOXC1*-gRNA6-F | TAGGTCTCCTCGACTGGTCGGGTTTTAGAGCTAGAA |
| *HvLOXC1*-gRNA6-R | CGGGTCTCATCGAAGGTGACGTGCACCAGCCGGG |
| OsU3-F | AGTACCACCTCGGCTATCCACA |
| UGW-gRNA-R | GGACCTGCAGGCATGCACGCGCTAAAAACGGACTAGC |

**Table S4** PTG sequences in the two editing constructs.

| **Vector** | **Gene (Architecture)** | **Sequence (5′-3′)** |  |
| --- | --- | --- | --- |
| *PTG-HvLOXB*  */Cas9* | PTG1 (tRNA+gRNA3+  tRNA+gRNA4) | GATCCGTGGCAACAAAGCACCAGTGGTCTAGTGGTAGAATAGTACCCTGCCACGGTACAGACCCGGGTTCGATTCCCGGCTGGTGCACACGACCGGAGAGTACAAGGGTTTTAGAGCTAGAAATAGCAAGTTAAAATAAGGCTAGTCCGTTATCAACTTGAAAAAGTGGCACCGAGTCGGTGCAACAAAGCACCAGTGGTCTAGTGGTAGAATAGTACCCTGCCACGGTACAGACCCGGGTTCGATTCCCGGCTGGTGCACTTCCTTGGGTACTCTCTCAGTTTTAGAGCTAGAAATAGCAAGTTAAAATAAGGCTAGTCCGTTATCAACTTGAAAAAGTGGCACCGAGTCGGTGCTTTTTTTTT |  |
|  |  |  |  |
| *PTG-HvLOXC1*  */Cas9* | PTG2 (tRNA+gRNA5+  tRNA+gRNA6) | GATCCGTGGCAACAAAGCACCAGTGGTCTAGTGGTAGAATAGTACCCTGCCACGGTACAGACCCGGGTTCGATTCCCGGCTGGTGCACGGCATCGTGAGCGACCTGAGTTTTAGAGCTAGAAATAGCAAGTTAAAATAAGGCTAGTCCGTTATCAACTTGAAAAAGTGGCACCGAGTCGGTGCAACAAAGCACCAGTGGTCTAGTGGTAGAATAGTACCCTGCCACGGTACAGACCCGGGTTCGATTCCCGGCTGGTGCACGTCACCTTCGACTGGTCGGGTTTTAGAGCTAGAAATAGCAAGTTAAAATAAGGCTAGTCCGTTATCAACTTGAAAAAGTGGCACCGAGTCGGTGCTTTTTTTTT |  |
| *PTG-HvLOXAC1 /Cas9* | PTG3 (tRNA+gRNA1+  tRNA+gRNA2+  tRNA+gRNA5+  tRNA+gRNA6) | GATCCGTGGCAACAAAGCACCAGTGGTCTAGTGGTAGAATAGTACCCTGCCACGGTACAGACCCGGGTTCGATTCCCGGCTGGTGCACCTCAAGACGTCCGACTTCCGTTTTAGAGCTAGAAATAGCAAGTTAAAATAAGGCTAGTCCGTTATCAACTTGAAAAAGTGGCACCGAGTCGGTGCAACAAAGCACCAGTGGTCTAGTGGTAGAATAGTACCCTGCCACGGTACAGACCCGGGTTCGATTCCCGGCTGGTGCAAGTCTGGACCCTAGCAAGTTGTTTTAGAGCTAGAAATAGCAAGTTAAAATAAGGCTAGTCCGTTATCAACTTGAAAAAGTGGCACCGAGTCGGTGCAACAAAGCACCAGTGGTCTAGTGGTAGAATAGTACCCTGCCACGGTACAGACCCGGGTTCGATTCCCGGCTGGTGCACGGCATCGTGAGCGACCTGAGTTTTAGAGCTAGAAATAGCAAGTTAAAATAAGGCTAGTCCGTTATCAACTTGAAAAAGTGGCACCGAGTCGGTGCAACAAAGCACCAGTGGTCTAGTGGTAGAATAGTACCCTGCCACGGTACAGACCCGGGTTCGATTCCCGGCTGGTGCACGTCACCTTCGACTGGTCGGGTTTTAGAGCTAGAAATAGCAAGTTAAAATAAGGCTAGTCCGTTATCAACTTGAAAAAGTGGCACCGAGTCGGTGCTTTTTTTTT |  |
|  |  |  |  |

Pre-tRNA is shown in blue font; the gRNA spacer is shown in the red font; the gRNA scaffold is shown in green font; TTTTTTTTT is Pol III terminator.

**Table S5** Primers for the amplification of partial genomic sequence.

| **Gene** | **Forward primer (5′-3′)** | **Reverse primer (5′-3′)** | **Product size (bp)** |
| --- | --- | --- | --- |
| *HvLOXA* | CAACTCCGACCACCCTTACC | CGTGGCGTAGATGAAGTTGC | 929 |
| *HvLOXB* | CCACAACAAATCTACCGTCTC | ACCTCCAGCAATCCAGTAAG | 776 |
| *HvLOXC1* | ATAAATTGGCCTCCCGGTCG | GAGTTGGCGACGAAGACGAT | 798 |
